# Food-Specific Decentering Experiences Are Associated with Reduced Food Cravings in Meditators: A Preliminary Investigation

**DOI:** 10.1007/s12671-016-0554-4

**Published:** 2016-07-05

**Authors:** Esther K. Papies, Martine van Winckel, Mike Keesman

**Affiliations:** 1 Institute of Neuroscience and Psychology, University of Glasgow, 58 Hillhead Street, Glasgow, G12 8QB UK; 2 Department of Psychology, Utrecht University, Utrecht, The Netherlands

**Keywords:** Mindfulness, Mindful attention, Decentering, Vipassana, Food, Craving, Desire, Meditation, Self-regulation

## Abstract

This study examined the association of food-specific decentering experiences with food cravings in a sample of meditators. Decentering refers to viewing one’s thoughts as transient mental events and thus experiencing them as less subjectively real. This process has been suggested to be a key mechanism underlying the effects of mindfulness and many contemplative practices. Although most earlier studies have focused on the effects of decentering with regard to negative affect, some studies have shown that brief inductions of decentering among non-meditators reduce food cravings as well as unhealthy food choices. Here, we report a preliminary investigation of whether the food-specific decentering experiences that meditators have in daily life are associated with fewer food cravings. A small sample of meditators (*N* = 33, female = 15) answered a number of questions about decentering experiences with regard to thoughts about food, and they completed the short version of the Food Cravings Questionnaire–Trait and a measure of meditation experience. Results confirmed that both more meditation experience and more food-specific decentering experiences were associated with fewer food cravings in daily life. In addition, results suggested that when participants had stronger decentering experiences, they experienced fewer food cravings, regardless of their level of meditation experience. Exploratory analyses further revealed that decentering was more strongly associated with reduced cravings in women than in men. These preliminary findings suggest that food-specific decentering experiences indeed help meditators deal with food desires, and thus extend the evidence for decentering effects into the domain of reward. Future research might investigate this in larger samples, validate a food-specific measure of decentering, and consider the broader implications of decentering experiences in daily life.

**Electronic supplementary material:**

The online version of this article (doi:10.1007/s12671-016-0554-4) contains supplementary material, which is available to authorized users.

## Introduction

Over the past decade, we have witnessed a strong increase in mindfulness-based interventions to facilitate self-regulation. In addition to the original mindfulness-based stress reduction program (Kabat-Zinn 1982), mindfulness has been integrated, for example, into programs for quitting smoking, alcohol consumption, and drug use (e.g., Bowen and Marlatt 2009; Brewer et al. 2011; Tapper et al. 2009; Witkiewitz et al. 2013). In the domain of eating behavior, a growing number of programs are integrating mindfulness skills to improve the regulation of healthy eating, among both clinical and non-clinical populations (e.g., Alberts et al. 2012; Daubenmier et al. 2011; Kristeller et al. 2014; Timmerman and Brown 2012). Increasingly, however, researchers have become interested not only in the effectiveness of mindfulness, but also in the underlying psychological mechanisms by which it affects cognitive and behavioral outcomes (e.g., Hölzel et al. 2011; Kang et al. 2013; Karremans et al. 2015; Papies 2016). It has been suggested, for example, that the mindfulness component of decentering, or viewing one’s thoughts and cravings as mere mental events, is highly effective to help deal with craving and desire and thus to facilitate self-regulation. This perspective is trained in mindfulness programs, but is also part of most contemplative practices (e.g., Desbordes et al. 2014; Dunne 2011, 2015). However, little is known about the effects of this perspective on meditators’ daily life experiences.

A number of models of mindfulness have recently been suggested in the psychological literature, specifying the multidimensional nature of this construct and its working mechanisms (e.g., Bishop et al. 2004; Hölzel et al. 2011; Kang et al. 2013; Shapiro et al. 2006). Although these models place different emphases, they seem to agree on two central components of mindfulness. The first of these is the regulation of the focus and stability of attention, which is typically directed and maintained on one’s present-moment experience (e.g., Bishop et al. 2004). The second component is an attitude of openness and acceptance of one’s experiences, based on the insight that one’s thoughts and experiences are transient mental events, rather than stable reflections of reality (Bishop et al. 2004; Teasdale 1999). This can be achieved by learning to observe one’s thoughts and experiences from a metacognitive perspective as events that arise in the mind, in contrast to being immersed in them as usual and experiencing them as “real” (Papies et al. 2012). This metacognitive insight is often referred to as decentering, and it is of major importance in meditative practice (e.g., Dreyfus 2011; Dunne 2011; Thrangu Rinpoche 2004). Indeed, decentering scores have been shown to be increased among participants with more experience in mindfulness and other types of meditation (Lau et al. 2006).

A number of studies have pointed toward the effectiveness of decentering for adaptive cognition and behavior and for mental health (for a recent review, see Bernstein et al. 2015). Research evaluating the benefits of mindfulness-based cognitive therapy, in which decentering plays a crucial role, has shown that including mindfulness into treatment for depression can significantly reduce relapse in depression (Teasdale et al. 2000). Metacognitive awareness and decentering may be important mechanisms for these effects (Bieling et al. 2012; Carmody et al. 2009; Fresco et al. 2007b; Teasdale et al. 2002). In the domain of stress, a recent neuroimaging study demonstrated how decentering causes a shift in perspective away from self-immersed processing of stressful events (Lebois et al. 2015). In addition, decentering scores after mindfulness training have been shown to predict reduced stress and psychiatric symptoms (Lau et al. 2006).

Recent research supports the idea that decentering may also be crucial for the effects of mindfulness in other areas of self-regulation, particularly in areas where positive affect and reward expectations, rather than negative affect, lead to maladaptive cognition and behavior. Specifically, recent experimental studies among non-meditators have shown that inducing a decentered perspective toward tempting stimuli can reduce desire for them. In one series of experiments (Papies et al. 2012; Papies et al. 2015), participants first learned to view their thoughts in response to images of tempting foods as mere mental events. Then, they were exposed to the same food pictures again, and their implicit approach responses, their desire toward these foods, as well as their food cravings and choices were assessed. Across several experiments, having viewed tempting foods from a decentered perspective reduced their motivational salience and facilitated healthy choices, compared to various control conditions in which participants did not apply a decentered perspective. In a similar experiment, applying a cognitive defusion strategy of viewing one’s chocolate-related thoughts as transient and as separate from oneself reduced the number of chocolates research participants consumed over a 5-day period (Jenkins and Tapper 2014). In a neuroimaging study where smokers applied a similar decentering and acceptance strategy to images of cigarettes, this reduced experienced craving as well as craving-related neural activity (Westbrook et al. 2013).

Together, the findings described above suggest that experimentally inducing a decentered perspective effectively reduces cravings among non-meditators. To date, however, there is no empirical evidence as to the effects of decentering experiences on cravings among active meditators. In other words, do meditators have decentering experiences in their daily lives that help them deal with cravings, for example in the domain of food? Previous research has shown that especially unhealthy, high-calorie food triggers spontaneous re-experiences of eating and enjoying it, based on earlier behavior (e.g., Papies 2013; Simmons et al. 2005), and it has been suggested that these eating and reward simulations contribute to the development of craving and desire (Papies and Barsalou 2015). Applying decentering to food-related thoughts and thus seeing one’s food-related thoughts as transient mental events might change the perspective of the reward simulations actually happening to one’s self. In other words, applying decentering might make the reward simulations less subjectively real, thus preventing the development of full-blown craving and desire. Indeed, experimental studies demonstrate the effectiveness of decentering for reducing food cravings when participants were trained to adopt a decentered perspective with regard to the thoughts that were triggered by the tempting food items (Jenkins and Tapper 2014; Papies et al. 2012, 2015). Similar effects might occur in active meditators as a result of their spontaneous, food-specific decentering experiences.

In addition, since the attention training of meditative practice may help to regulate one’s attention away from tempting stimuli and thoughts, it might be that among active meditators, those individuals with more meditation experience have fewer food cravings overall. However, the benefits of decentering might be relatively independent of extended meditation practice. Decentering is related to the insight into the nature of one’s thoughts as transient mental events (Dreyfus 2011; Dunne, 2015) and has been shown to affect responses to food cues in non-meditators after brief training sessions. Thus, in contrast to the cumulative benefits of attention training, food-specific decentering might be effective for reducing cravings independent of one’s lifetime history of meditation. Meditation experience has been suggested, however, to increase the awareness of one’s internal experiences and may thus increase awareness of one’s thoughts about food. Although such awareness may be useful for successfully applying decentering to food-related thoughts, little research so far has explored this.

The aim of current correlational study was to provide an initial, preliminary assessment of the effect of spontaneous decentering from food-related thoughts, in order to start examining whether adopting this perspective in one’s daily life outside the laboratory would effectively prevent the development of food cravings, similar to laboratory settings. Our hypotheses were, first and foremost, that food-specific decentering experiences would be negatively associated with food cravings. In addition, while we hypothesized that individuals with more meditation experience would experience fewer food cravings overall, we expected the benefits of decentering to be relatively independent of extended meditation practice. In other words, we predicted that participants with high food-specific decentering scores would have relatively few food cravings, regardless of their lifetime meditation experience. In addition to these central hypotheses, we explored whether more meditation experience is related to more frequent decentering experiences and to increased awareness of craving-related thoughts. To test these hypotheses, we used a novel, yet unvalidated measure to assess food-specific decentering, which consisted of a number of questions adapted from an existing general decentering questionnaire (Fresco et al. 2007a) and from training procedures successfully used to induce decentering in previous studies (Papies et al. 2012, 2015). We also constructed three questions to assess awareness of one’s food-related thoughts, which were included merely to explore whether awareness increases with meditation experience. These questions on food-specific decentering and awareness of food thoughts, together with established measures of food cravings and of meditation experience, were presented to a sample of active meditators in order to assess the association of food-specific decentering experiences with food cravings.

## Method

### Participants

Thirty-three meditation practitioners (15 female), ages 20–80 (*M* = 44.06, *SD* = 16.52) from local meditation communities in Atlanta, GA (USA) participated. Participants were recruited by visiting a number of meditation centers from different traditions (Tibetan Buddhism, Shambhala, Zen, Vipassana). Accordingly, participants in this study practiced in several styles and traditions, such as Shamatha/breath-focus, Vipassana/insight, compassion, and zazen. Several subjects had experience in multiple traditions, as is common with Western lay practitioners. Twenty-one (63.6 %) of the participants were Caucasian or White; 8 (24.2 %) were Asian; 2 (6.0 %) were African American; one participant was Arab and one was Indian. Their mean BMI was 23.18 (*SD* = 4.65; range 15.4–41.7).

### Procedure

Participants could complete the paper version of the questionnaire during recruitment. Alternatively, they could register their email address to receive a link that directed them to the Web site on which they could complete the online version of the questionnaire at a later time. A number of meditation centers also posted a link to the study on their Web site. The study was introduced as “a study on meditation and experiences with food,” and we did not specify dieting, wanting to reduce food cravings, or a specific level of meditation experience as a condition for participating. Participants first completed an informed consent form, followed by the questionnaire, which took approximately 5–10 min to complete. Participants were free to skip any of the questions that they did not want to answer. The study period lasted 3 weeks, in which 40 individuals started the online study from which 26 complete datasets were recorded. Seven participants completed the paper and pencil questionnaire.

### Measures

#### Decentering from Food Thoughts

To assesses food-specific decentering, we used a number of questions from the “Experiences Questionnaire” that we adjusted to refer specifically to food (“I am able to separate myself from my thoughts about food,” “I can distance myself from my thoughts about food,” “I get lost in my thoughts about food (recode)”; see Fresco et al. 2007a). In addition, we included two items that were modeled on existing instructions to induce decentering in previous experiments (“When I have thoughts about food, I notice these thoughts come and go,” “I consider my thoughts about food as transient events in my mind”; Papies et al. 2012, 2015). Furthermore, we included three new items to assess the potential phenomenological effects of decentering from one’s food-related thoughts (“The thoughts I have about food are very intense,” “The thoughts I have about food seem very real,” “Food affects me strongly,” all recoded; see Table 1). A number of the questions used were included in an initial validation study, which found that these loaded on a “decentering” factor correlating negatively with food cravings, and that participants scored higher on these questions when they had completed a brief decentering training compared to a control condition (Van Winckel 2014). All questions included in the current study were assessed by members of two different labs with expertise in mindfulness research with regard to wording, comprehensibility, and face validity.

**Table 1:** Questions used to assess awareness of and decentering from food-related thoughts

*Construct*	*Item*
Awareness of food-related thoughts	1. I notice that food elicits certain reactions in me.
	2. I notice what I think about food.
	3. I notice how I react to food.
Decentering from food-related thoughts	1. When I have thoughts about food, I notice these thoughts come and go.
	2. I consider my thoughts about food as transient events in my mind.
	3. The thoughts I have about food are very intense. (recode)
	4. I get lost in my thoughts about food. (recode)
	5. The thoughts I have about food seem very real. (recode)
	6. Food affects me strongly. (recode)
	7. I can distance myself from my thoughts about food.
	8. I am able to separate myself from my thoughts about food.

Participants were instructed to “answer the following questions with regard to how you relate to your thoughts about food in your daily life” and to “indicate to what extent these statements are typically true for you.” They were also reminded that there were no right or wrong answers. All questions were answered on a 7-point scale ranging from “1–Never true for me” to “7–Always true for me.” Across the questionnaire, we formulated a number of items to be recoded (i.e., where higher scores indicate less decentering) in order to stimulate participants to consider and answer each item carefully.

The resulting scale demonstrated good internal reliability (α = 0.84). The scale as a whole, however, was not subjected to systematic validation in the current study.

#### Awareness of Food Thoughts

We also included three items to measure the awareness of one’s food-related thoughts and experiences (“I notice that food elicits certain reactions in me,” “I notice what I think about food,” “I notice how I react to food”; Cronbach’s α = 0.68”). All questions were answered on a 7-point scale ranging from “1–Does not apply to me at all” to “7–Applies to me very much.”

#### Food Cravings

To assess trait food cravings, we administered the 15-item Trait Food Craving Questionnaire (FCQ-T-r; Meule et al. 2014; α = 0.95), which is a shortened version of the 39-item Food Craving Questionnaire–Trait (Cepeda-Benito et al. 2000) with items such as “I have no willpower to resist my food crave,” “My emotions often make me want to eat,” “It is hard for me to resist the temptation to eat appetizing foods that are in my reach,” “I feel like I have food on my mind all the time.” Participants indicated whether this was “never,” “rarely,” “sometimes,” “often,” “usually,” or “always” true for them.

#### Lifetime Meditation Experience

Participants were asked to indicate how often they currently meditated, how long they usually meditated, and how many years of experience they had of meditating at this rate. They were then asked to describe the frequency of any practice they had before their current practice, to describe any retreats they had done, and to describe the style of meditation they were currently practicing. These questions served to estimate lifetime practice hours by means of the procedure described by Hasenkamp and Barsalou (2012), see Supplementary Materials. Participants were also asked to indicate how long ago they had last meditated. Finally, participants reported their age, height and weight, race/ethnicity, and gender.

### Data Analyses

We first computed bivariate correlations between the study variables (food craving, food-specific decentering, awareness of food thoughts, meditation experience) in order to explore their relationships, particularly the relation of awareness of food thoughts and meditation experience, which we had only included for exploratory reasons. For our main analyses, we conducted regression analyses with mean-centered predictor variables. We adopted the recommendations of Aiken and West (1991) to decompose interactions in regression analyses by using the regression model to estimate the simple slopes of one predictor variable at specific values of the other (typically one SD below and one SD above the mean, respectively) and then testing whether each simple slope is different from zero.

## Results

Table 2 displays the frequency and duration of participants’ current meditation practice and the number of retreats completed, showing that about half of participants meditated daily or almost daily and had completed one or several retreats. Using the procedure introduced by Hasenkamp and Barsalou (2012), meditation experience ranged from 3.1 to 12,558 h. One participant scored more than 3 SD above the mean of lifetime meditation experience (12,558 h) and was therefore not included in the analyses. The average hours of estimated lifetime meditation practice across the remaining group (*N* = 32) was 1199 h (*SD* = 1638 h). Overall, participants scored close to the midpoint of the scale for both food-specific decentering (*M* = 4.28, *SD* = 1.13) and food cravings (*M* = 2.51, *SD* = .83) and scored above the midpoint for awareness (*M* = 5.17, *SD* = 1.05).

**Table 2:** Frequency and duration of current meditation practice and number of retreats completed by participants

*Meditation frequency*	*Frequency (%)*
Daily or almost daily	18 (54.4 %)
2–4 days per week	10 (30.3 %)
Approx. 1 day per week	2 (6.1 %)
Less than 1 day per week	3 (9.1 %)
*Meditation duration*	
More than 30 min per day	11 (33.3 %)
15–30 min per day	16 (48.5 %)
5–15 min per day	6 (18.2 %)
*Retreats*	
None	9 (27.2 %)
One	2 (6.1 %)
Several	19 (57.6 %)
No information	3 (9.1 %)

Table 3 displays bivariate correlations among food cravings, food-specific decentering, awareness of food thoughts, and meditation experience. Food cravings were negatively correlated with food-specific decentering and with meditation experience. Meditation experience did not correlate significantly with decentering from food thoughts, or with awareness of food thoughts. Thus, among this sample of meditators, those with more experience did not have more food-specific decentering experiences or more awareness of their food-related thoughts than those with less experience.

**Table 3:** Bivariate correlations among food cravings (Meule et al. 2014), food-specific decentering, awareness of food thoughts, and lifetime meditation experience

	Food-specific decentering	Awareness of food thoughts	Meditation experience
Food cravings	−0.62^a^	0.27	−0.47 ^a^
Food-specific decentering		−0.29	0.25
Awareness of food thoughts			−0.20

^a^Correlation is significant at *p* < 0.001

In order to test our hypothesis that food-specific decentering experiences are associated with fewer food cravings among meditators, we first conducted a regression analysis in which we entered food-specific decentering scores as the sole predictor of food cravings. This confirmed that higher food-specific decentering scores were associated with fewer cravings, β = −0.62, *t*(30) = −4.31, *p* < 0.001, *R*^2^ = 0.38.

An additional regression analysis with lifetime meditation experience as sole predictor of food cravings also revealed a significant effect, β = −0.47, *t*(30) = −2.89, *p* = 0.007, *R*^2^ = 0.22, showing that more meditation experience was associated with fewer cravings in daily life. This effect remained when we controlled for participant age, β = −0.49, *t*(29) = −2.98, *p* = 0.006, *R*^2^ = 0.24, while age itself was not a significant predictor of cravings, *p* = 0.38.

**Fig. 1:**
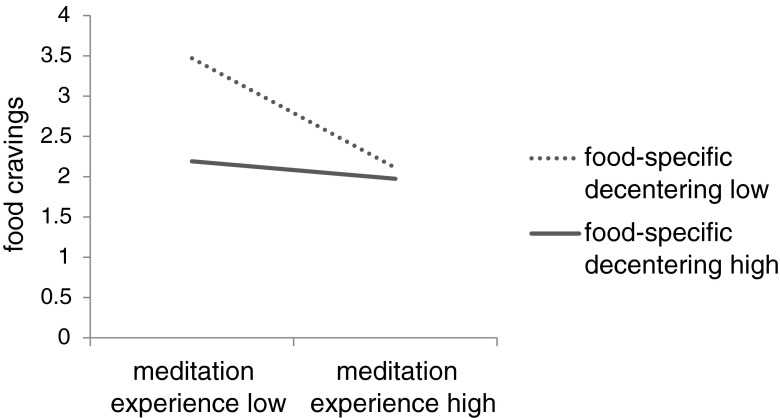
The association of meditation experience with food cravings at different levels of food-specific decentering. High and low values denote one standard deviation above and below the mean of the scale, respectively (see Aiken and West 1991; meditation experience *M* = 1199 h, *SD* = 1638 h; food-specific decentering *M* = 4.28, *SD* = 1.13)

Next, we examined the interaction of food-specific decentering and meditation experience in order to test our hypothesis that food-specific decentering experiences are particularly effective for reducing cravings, so that when decentering scores are high, participants experience few food cravings, even if they do not have high levels of meditation experience. We first entered the main effects of food-specific decentering and experience and then their interaction. Variance inflation factors were low (<1.4) suggesting that multicollinearity was not a concern. Both main effects remained significant (β = −0.43, *t*(28) = −3.02, *p* = 0.005; β = −0.48 , *t*(28) = −3.17, *p* = 0.004, respectively), and the marginally significant interaction term, β = −0.30, *t*(28) = 1.99, *p* = 0.057, Δ*R*^2^ = 0.06, confirmed that the effect of meditation experience was indeed somewhat different for participants with low vs high food-specific decentering scores. This effect is illustrated in Fig. 1. We decomposed this interaction in line with the recommendations of Aiken and West (1991) and examined the simple slopes of meditation experience at relatively high and relatively low levels of decentering (one SD above and below the mean, respectively).

In line with our hypothesis, these analyses revealed that while meditation experience was associated with decreased food cravings among participants with relatively low food-specific decentering scores, β = −0.82, *t*(28) = −2.94, *p* = 0.007, meditation experience was not associated with reduced food cravings among participants with high food-specific decentering scores, β = −0.13, *t*(28) = −0.79, *p* = 0.44. Indeed, as we have seen above and as displayed below, these meditators had fewer food cravings in general. In other words, when participants had strong food-specific decentering experiences, they experienced fewer food cravings, regardless of their level of meditation experience.

Finally, we explored whether the effect of decentering on cravings was different for men and women by conducting a regression analysis which included food-specific decentering, gender, and their interaction. While gender did not have a main effect on cravings, it did moderate the effect of food-specific decentering, β = −0.93 , *t*(28) = −2.25, *p* = 0.03, Δ*R*^2^ = 0.09. Simple slope analyses revealed that food-specific decentering was more strongly associated with reduced food cravings among women, β = −0.83, *t*(13) = −5.37, *p* < 0.001, than among men, β = −.46, *t*(15) = −2.03, *p* = 0.06.

## Discussion

The findings of the present study suggest that food-specific decentering is strongly associated with reduced food cravings. We assessed the degree to which meditators experienced decentering from food-related thoughts, as well as their meditation experience and their food cravings in daily life. Given the small sample size and the experimental, unvalidated measure of decentering used, our findings should be interpreted with caution. In line with our predictions, however, we found that the degree to which meditators experienced decentering from food-related thoughts was negatively associated with food cravings. In addition, this association occurred even among those meditators with a relatively brief history of meditation practice—they experienced relatively few food cravings, as long as they reported strong food-specific decentering experiences. Albeit preliminary, this suggests that the effect of food-specific decentering experiences for reducing cravings is not enhanced by more extended practice, which is in line with the conceptualization of decentering as an insight component of mindfulness. At the same time, our results also showed that meditation experience itself predicts reduced craving, such that participants with more practice had fewer food cravings in daily life, even if they did not report strong food-specific decentering experiences. Thus, extended meditation practice and food-specific decentering have different and unique associations with craving in daily life. The underlying mechanisms of these effects and their interplay should be examined in more detail in future studies with a larger sample. In addition, a systematic validation of domain-specific decentering measures will be necessary to strengthen the conclusions we can draw from such findings.

Exploratory analyses revealed that the negative association between food-specific decentering and food cravings was particularly pronounced among women, compared to men. This finding likely reflects the general tendency among women to be more preoccupied with food and body weight (e.g., Wardle et al. 2006). As a result, a decentered perspective can be applied to a particularly rich and compelling set of food-related thoughts, making this perspective particularly effective. Such an effect would parallel the finding that learning to apply decentering to one’s food-related thoughts is particularly effective when motivational states are strong, for example when an individual is hungry and thus more preoccupied with food (Papies et al. 2015). Further research should address this gender effect more directly along with its underlying mechanisms.

The current study found no significant correlation between meditation experience and food-specific decentering. This could be due to the relatively small sample. However, the correlation coefficient was also small, which suggests that longer durations of contemplative practice did not markedly strengthen decentering experiences related to food. Possibly, some meditators incorporate applying a decentered perspective heavily into their practice, while for others it is less central, and meditating more does not seem to strengthen the experience of decentering from food-related thoughts in daily life. Future research could examine whether some meditators maintain a contemplative practice for reasons unrelated to decentering and its potential benefits, and also whether some meditators apply a decentered perspective in some realms of experience, but not in the realm of craving and desire, and to what degree this is motivated by individual differences in preoccupations with food.

This finding again corresponds to the conceptualization of decentering as a conceptual insight into the nature of one’s thoughts, which is not per se strengthened by extended practice. Indeed, decentering has been shown to affect non-meditators’ cognition and behavior after very brief training (e.g., Jenkins and Tapper 2014; Lacaille et al. 2014; Papies et al. 2012, 2015) and through mindfulness training (Lau et al. 2006). However, we do assume that meditation practice and especially the attention training component of meditation will support the consistent and effective application of a decentered perspective in relevant situations and domains, and thus allow practitioners to fully reap its benefits in daily life (see also Dreyfus 2011; Papies et al. 2015).

Naturally, high awareness of one’s food-related thoughts is important for this, since these thoughts can develop into cravings when one gets immersed in them too much (Kavanagh et al. 2005; Papies et al. 2015). The current study found that meditators’ awareness of food-related thoughts was high (i.e., well above the midpoint of the scale), and it found no correlation between meditation experience and awareness. Future research could examine the effects of meditation practice on awareness in various domains of reward in more detail.

In addition to these findings, we would like to point out three important limitations of the current study. First of all, the current study is merely preliminary, due to the small sample, and because we only included an experimental and so-far unvalidated measure of food-specific decentering. Although the findings were promising, these features warrant a replication study with a larger sample, which should also validate the decentering measure before it can be used in further research. In addition, such a study might include a measure of general decentering, to assess the degree to which food-specific decentering builds on this skill, and possibly has unique effects beyond it.

Second, the partial online nature of the study did not allow us to tightly control who participated. Although we have little reason to assume that this caused any problems, more control over one’s sample would be desirable. Finally, a third potential problem concerns the self-report nature of the measures we used. As Grossman and van Dam (2011) have pointed out, self-report measures designed to assess mindfulness or related constructs potentially have a number of problems. To mention a few, questions may be interpreted differently by participants with and without mindfulness experience, participants with mindfulness training may provide “better” answers to justify their extensive investments in time and effort, and participants’ insights into the issues assessed may be changing dynamically with increasing meditation experience. In the current study, however, only active meditators were included, which makes it more likely that they interpreted the questions rather consistently, especially because most of the meditation practices that participants described seem to include decentering elements. In addition, the fact that all participants were meditators makes it less likely that they provided biased answers to “justify” their investments in meditation practice. Indeed, participants’ lifetime meditation experience did not correlate strongly with food-specific decentering scores, which further suggests that this aspect of self-report measures may not be a concern here.

In general, however, we strongly advocate the use of measures that do not rely on self-report to assess decentering and its effects. Earlier research has shown that decentering applied to one’s thoughts about food pictures reduced implicit approach impulses toward tempting food, and it reduced unhealthy food choices when participants were not aware that their behavior was being observed. This suggests that decentering from desire thoughts affects implicit measures of desire (Papies et al. 2012, 2015). Although it would be desirable to employ similarly implicit measures of decentering experiences themselves, these do not currently exist, and developing such measures remains an important direction for further study.

Neuroimaging techniques provide initial demonstrations of how decentering from one’s own initial responses changes the processing of negative and of reward-related stimuli (Hölzel et al. 2013; Lebois et al. 2015; Westbrook et al. 2013). These insights could further inform behavioral measures of decentering in various domains. In addition, future research could examine whether the neural and cognitive mechanisms are the same in novices, who apply decentering to their experiences for the first time, and in experienced meditators, who may apply decentering more automatically. We also suggest that it will be particularly important to further develop the study of decentering in the domain of problematic craving and desires, such as for food, alcohol, sex, and other rewarding stimuli. Currently, our insight in decentering effects is very limited in these areas. However, reward-related processes can strongly impact well-being, as both the current scientific literature as well as Buddhist writings on the effects of craving and attachment point out (see Ricard 2007; Wallace and Shapiro 2006), making this an important area for studying and potentially applying decentering.

## Electronic supplementary material

Below is the link to the electronic supplementary material.

ESM 1 (DOCX 44 kb)
