# Supplementary material for: Food-Specific Decentering Experiences Are Associated with Reduced Food Cravings in Meditators: A Preliminary Investigation
Source: Mindfulness (N Y). 2016 Jul 5;7(5):1123–31. doi: 10.1007/s12671-016-0554-4 (PMC5010617; doi:10.1007/s12671-016-0554-4)
Supplement: Supplementary file 1 — (DOCX 44 kb) [file 12671_2016_554_MOESM1_ESM.docx]

**Supplementary Materials.**

**Estimating lifetime meditation experience**

Lifetime meditation experience was estimated as described by Hasenkamp & Barsalou (2012). Participants were asked to report their current meditation practice using three variables: days/week (*A*), minutes/day (*B*), and years at this frequency (*C*). Values were assigned to participants’ responses as described below. Participants were also asked if there was another time that they had practiced a different style or at a different frequency. If so, they repeated their answers for the previous practice to yield the values *D*, *E*, and *F*. Finally, they were asked to list any meditation retreats they had attended, and for each, the number of days and duration per day of practice. The number of days was multiplied by the duration and summed over retreats to yield the value *R*. Total lifetime hours was then calculated as follows:

Days/week (assign value *A*, and *D* if present)

- Daily or almost daily = 6
- 2–4 days per week = 3
- Approximately 1 day per week = 1
- Less than one day per week = 0.5

Minutes/day (assign value *B*, and *E* if present)

- 5–15 minutes per day = 0.12
- 15–30 minutes per day = 0.33
- More than 30 minutes per day = 0.67

Years at this frequency (assign value *C*, and *F* if present)

Retreats (assign value *R*)

- Days × hours/day, summed over retreats. One day was calculated as including eight hours of meditation. Answers like ‘several’, ‘now and then’, and ‘multiple’ were coded as ‘5 times’. If participants only responded with ‘yes’ but did not provide details about duration or frequency, the retreats were not included and coded as ‘0’.

The formula for estimated lifetime hours was: Hours = [*A* × 52 × *B* × *C*] + [*D* × 52 × E × *F*] + *R* with *D*, *E*, and *F* included only if there are values.


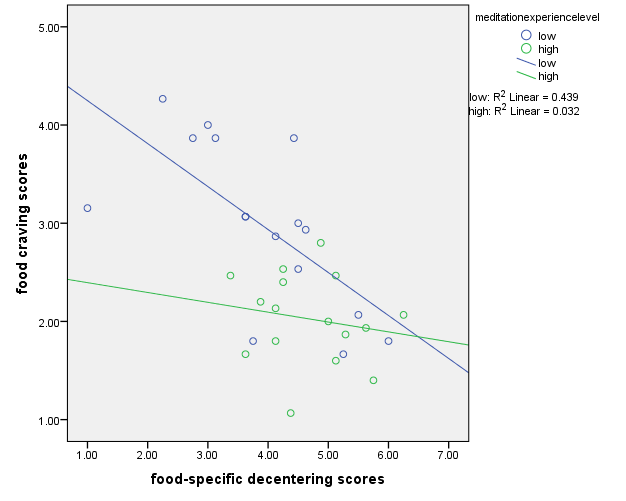


Supplementary Figure.

Scatterplot (N = 32) displaying food-specific decentering and food-craving scores for participants with relatively low and relatively high levels of mediation experience (median split; median = 345 hours).
